# Supplementary material for: On the dynamic toroidal multipoles from localized electric current distributions
Source: Sci Rep. 2017 Aug 8;7:7527. doi: 10.1038/s41598-017-07474-4 (PMC5548821; doi:10.1038/s41598-017-07474-4)
Supplement: Supplementary file 1 — Supplementary Information [file 41598_2017_7474_MOESM1_ESM.pdf]

Supplementary Information for "On the dynamic toroidal multipoles from localized electric current distributions"

Ivan Fernandez-Corbaton*
Institute of Nanotechnology, Karlsruhe Institute of Technology, 76021 Karlsruhe, Germany

Stefan Nanz
Institut für Theoretische Festkörperphysik, Karlsruhe Institute of Technology, 76131 Karlsruhe, Germany

Carsten Rockstuhl
Institut für Theoretische Festkörperphysik, Karlsruhe Institute of Technology, 76131 Karlsruhe, Germany and Institute of Nanotechnology, Karlsruhe Institute of Technology, 76021 Karlsruhe, Germany

I. CONTRIBUTIONS TO b^ω_{jm}, a^ω_{jm} AND c^ω_{jm}

Equation (14) in [1] is an exact expression for the $\{a^\omega_{jm}, b^\omega_{jm}, c^\omega_{jm}\}$ coefficients in terms of integrals in both momentum and coordinate space. With $\mathbf{Q}_{jm}(\hat{\mathbf{p}})$ standing for any of the $\{\mathbf{X}_{jm}(\hat{\mathbf{p}}), \mathbf{Z}_{jm}(\hat{\mathbf{p}}), \mathbf{W}_{jm}(\hat{\mathbf{p}})\}$ and q^ω_{jm} for any of the corresponding $\{a^\omega_{jm}, b^\omega_{jm}, c^\omega_{jm}\}$, Eq. (14) in [1] reads

$$\sqrt{\frac{\pi}{2}} q^\omega_{jm} = \sum_{\bar{l}\bar{m}} (-i)^{\bar{l}} \int d\hat{\mathbf{p}}\ \mathbf{Q}^\dagger_{jm}(\hat{\mathbf{p}}) Y_{\bar{l}\bar{m}}(\hat{\mathbf{p}}) \int d^3\mathbf{r}\ \mathbf{J}_\omega(\mathbf{r}) Y^*_{\bar{l}\bar{m}}(\hat{\mathbf{r}}) j_{\bar{l}}(kr), \tag{1}$$

where $j_l(\cdot)$ are the spherical Bessel functions of the first kind.

As shown in [1, App. B], only terms with $\bar{l} = j$ contribute to the b^ω_{jm}, while the a^ω_{jm} and c^ω_{jm} get contributions from both $\bar{l} = j-1$ and $\bar{l} = j+1$. Explicitly:

$$\begin{aligned}
\sqrt{\frac{\pi}{2}} b^\omega_{jm} &= (-i)^j \sum_{\bar{m}=-j}^{\bar{m}=j} \int d\hat{\mathbf{p}}\ \mathbf{X}^\dagger_{jm}(\hat{\mathbf{p}}) Y_{j\bar{m}}(\hat{\mathbf{p}}) \int d^3\mathbf{r}\ \mathbf{J}_\omega(\mathbf{r}) Y^*_{j\bar{m}}(\hat{\mathbf{r}}) j_j(kr), \\
\sqrt{\frac{\pi}{2}} a^\omega_{jm} &= \\
&(-i)^{j-1} \sum_{\bar{m}=-(j-1)}^{\bar{m}=j-1} \int d\hat{\mathbf{p}}\ \mathbf{Z}^\dagger_{jm}(\hat{\mathbf{p}}) Y_{j-1\bar{m}}(\hat{\mathbf{p}}) \int d^3\mathbf{r}\ \mathbf{J}_\omega(\mathbf{r}) Y^*_{j-1\bar{m}}(\hat{\mathbf{r}}) j_{j-1}(kr) + \\
&(-i)^{j+1} \sum_{\bar{m}=-(j+1)}^{\bar{m}=j+1} \int d\hat{\mathbf{p}}\ \mathbf{Z}^\dagger_{jm}(\hat{\mathbf{p}}) Y_{j+1\bar{m}}(\hat{\mathbf{p}}) \int d^3\mathbf{r}\ \mathbf{J}_\omega(\mathbf{r}) Y^*_{j+1\bar{m}}(\hat{\mathbf{r}}) j_{j+1}(kr), \\
\sqrt{\frac{\pi}{2}} c^\omega_{jm} &= \\
&(-i)^{j-1} \sum_{\bar{m}=-(j-1)}^{\bar{m}=j-1} \int d\hat{\mathbf{p}}\ \mathbf{W}^\dagger_{jm}(\hat{\mathbf{p}}) Y_{j-1\bar{m}}(\hat{\mathbf{p}}) \int d^3\mathbf{r}\ \mathbf{J}_\omega(\mathbf{r}) Y^*_{j-1\bar{m}}(\hat{\mathbf{r}}) j_{j-1}(kr) + \\
&(-i)^{j+1} \sum_{\bar{m}=-(j+1)}^{\bar{m}=j+1} \int d\hat{\mathbf{p}}\ \mathbf{W}^\dagger_{jm}(\hat{\mathbf{p}}) Y_{j+1\bar{m}}(\hat{\mathbf{p}}) \int d^3\mathbf{r}\ \mathbf{J}_\omega(\mathbf{r}) Y^*_{j+1\bar{m}}(\hat{\mathbf{r}}) j_{j+1}(kr).
\end{aligned} \tag{2}$$

* ivan.fernandez-corbaton@kit.edu

II. CANCELLATION OF LONGITUDINAL FIELDS OUTSIDE THE SOURCE

We show that the longitudinal field with $|\mathbf{p}| = \omega/c$ is zero outside the source region.

We consider spatially confined monochromatic electric charge and current density distributions $\rho_\omega(\mathbf{r})$ and $\mathbf{J}_\omega(\mathbf{r})$ embedded in an isotropic and homogeneous medium with constant permittivity ϵ and permeability μ. We assume them to be confined in space. In the Lorenz gauge, the scalar and vector potentials meet the following inhomogeneous wave equations:

$$\begin{aligned}\left(\nabla^2 + (\omega/c)^2\right)\phi_\omega(\mathbf{r}) &= \frac{-\rho_\omega(\mathbf{r})}{\epsilon},\\ \left(\nabla^2 + (\omega/c)^2\right)\mathbf{A}_\omega(\mathbf{r}) &= -\mu\mathbf{J}_\omega(\mathbf{r}),\end{aligned} \tag{3}$$

where $c = 1/\sqrt{\epsilon\mu}$. Outside the source region, it can be shown that the spatial Fourier transforms of $\phi_\omega(\mathbf{r})$ and $\mathbf{A}_\omega(\mathbf{r})$ are non-zero only for $|\mathbf{p}| = \omega/c$ (see [1, App. A]). With the help of [2, Eq. (3.8)], they are readily seen to be proportional to the Fourier components of the sources in the same spherical shell domain:

$$\mathring{\phi}(\hat{\mathbf{p}}) = \frac{\mathring{\rho}_\omega(\hat{\mathbf{p}})}{4\pi\epsilon},\ \mathring{\mathbf{A}}(\hat{\mathbf{p}}) = \frac{\mu\mathring{\mathbf{J}}_\omega(\hat{\mathbf{p}})}{4\pi}. \tag{4}$$

In coordinate space, the electric field as a function of the potentials is

$$\mathbf{E}_\omega(\mathbf{r}) = i\omega\mathbf{A}_\omega(\mathbf{r}) - \nabla\phi_\omega(\mathbf{r}), \tag{5}$$

which, in momentum space ($\nabla \to i\mathbf{p}$) reads

$$\mathbf{E}_\omega(\hat{\mathbf{p}}) = i\omega\mathring{\mathbf{A}}(\hat{\mathbf{p}}) - i\mathbf{p}\mathring{\phi}(\hat{\mathbf{p}}). \tag{6}$$

The longitudinal electric field is hence

$$\mathbf{p}\cdot\mathbf{E}_\omega(\hat{\mathbf{p}}) = \mathbf{p}\cdot\left(i\omega\mathring{\mathbf{A}}(\hat{\mathbf{p}}) - i\mathbf{p}\mathring{\phi}(\hat{\mathbf{p}})\right). \tag{7}$$

Using Eq. (4) and that $\mathbf{p}$ is restricted to $\mathbf{p} = \frac{\omega}{c}\hat{\mathbf{p}}$ we can write Eq. (7) as

$$\begin{aligned}\frac{\omega}{c}\hat{\mathbf{p}}\cdot\mathbf{E}_\omega(\hat{\mathbf{p}}) &= \frac{\omega}{c}\hat{\mathbf{p}}\cdot\left(i\omega\frac{\mu\mathring{\mathbf{J}}_\omega(\hat{\mathbf{p}})}{4\pi} - i\mathbf{p}\frac{\mathring{\rho}_\omega(\hat{\mathbf{p}})}{4\pi\epsilon}\right)\\ &= \frac{i\omega\mu}{4\pi}\left(\frac{\omega}{c}\hat{\mathbf{p}}\cdot\mathring{\mathbf{J}}_\omega(\hat{\mathbf{p}}) - \omega\mathring{\rho}_\omega(\hat{\mathbf{p}})\right).\end{aligned} \tag{8}$$

The term inside the brackets in Eq. (8) is equal to zero because of the continuity equation in momentum space

$$\nabla\cdot\mathbf{J}_\omega(\mathbf{r}) = i\omega\rho_\omega(\mathbf{r}) \stackrel{\nabla\to i\mathbf{p}}{\longrightarrow} i\mathbf{p}\cdot\mathring{\mathbf{J}}_\omega(\hat{\mathbf{p}}) = i\omega\mathring{\rho}_\omega(\hat{\mathbf{p}}) \tag{9}$$

particularized at $|\mathbf{p}| = \omega/c$.

$$i\frac{\omega}{c}\hat{\mathbf{p}}\cdot\mathring{\mathbf{J}}_\omega(\hat{\mathbf{p}}) = i\omega\mathring{\rho}_\omega(\hat{\mathbf{p}}). \tag{10}$$

We conclude that, outside the source region, the longitudinal field with $|\mathbf{p}| = \omega/c$ produced by the current density exactly cancels the one produced by the charge density. Note that the result is gauge independent since it is a statement about the electric field.

III. THE SPLIT OF ELECTRIC AND TOROIDAL PARTS INTRODUCES OUT OF SHELL COMPONENTS IN BOTH OF THEM

In this appendix we show that the independent measurement of electric and toroidal parts is impossible.

Let us consider the expression of the exact frequency-dependent multipoles of electric parity a^ω_{jm} in Eq. (2). The monochromatic current $\mathbf{J}_\omega(\mathbf{r})$ appears in two different spatial integrals,

$$\int d^3\mathbf{r}\ \mathbf{J}_\omega(\mathbf{r})Y^*_{j-1\overline{m}}(\hat{\mathbf{r}})j_{j-1}(kr), \tag{11}$$

and

$$\int d^3\mathbf{r}\ \mathbf{J}_\omega(\mathbf{r})Y^*_{j+1\overline{m}}(\hat{\mathbf{r}})j_{j+1}(kr), \tag{12}$$

where $k = \omega/c$, $j_l(\cdot)$ are spherical Bessel functions, $r = |\mathbf{r}|$, $\hat{\mathbf{r}} = \mathbf{r}/|\mathbf{r}|$, and $Y_{ln}(\cdot)$ are scalar spherical harmonics.

Let us now split Eq. (11) into two parts by means of the small argument expansion of $j_{j-1}(kr)$. We isolate the first term of the expansion, which is of order $(kr)^{j-1}$ and obtain:

$$\begin{aligned}&\int d^3\mathbf{r}\ \mathbf{J}_\omega(\mathbf{r})Y^*_{j-1\overline{m}}(\hat{\mathbf{r}})j_{j-1}(kr) =\\ &\int d^3\mathbf{r}\ \mathbf{J}_\omega(\mathbf{r})Y^*_{j-1\overline{m}}(\hat{\mathbf{r}})\frac{(kr)^{j-1}}{[2(j-1)+1]!!} +\\ &\int d^3\mathbf{r}\ \mathbf{J}_\omega(\mathbf{r})Y^*_{j-1\overline{m}}(\hat{\mathbf{r}})\left\{j_{j-1}(kr) - \frac{(kr)^{j-1}}{[2(j-1)+1]!!}\right\},\end{aligned} \tag{13}$$

where $n!! = n(n-2)(n-4)\ldots$ is the double factorial.

As we will now show, this is the split that gives rise to the electric and toroidal parts in the original literature [3, 4]. The first term in Eq. (13) corresponds to the electric part, and the second term is contained in the toroidal part. The toroidal part also contains the whole contribution of the integrals involving $j_{j+1}(kr)$ in Eq. (12).

Let us now see this splitting in the original literature [3, 4]. We start from the definition of the time-dependent exact multipoles of electric parity, which can be written from Eqs. 20 and 24 in Ref. 3 (also from Eqs. 1.3 and 1.10 in Ref. 5):

$$a_{jm}(k,t) = \int d^3\mathbf{r} \left[\sqrt{\frac{j+1}{2j+1}} j_{j-1}(kr)\mathbf{Y}_{j,j-1,m}(\hat{\mathbf{r}}) + \sqrt{\frac{j}{2j+1}} j_{j+1}(kr)\mathbf{Y}_{j,j+1,m}(\hat{\mathbf{r}})\right]^\dagger \mathbf{J}(\mathbf{r},t), \quad (14)$$

where $\mathbf{Y}_{jlm}(\cdot)$ are vector spherical harmonics.

The split between electric and toroidal parts can be seen in Eq. 38 of Ref. 4, and Eq. 4.6 of Ref. 4:

$$a_{jm}(k,t) = \partial_t Q_{jm}(0,t) + k^2 T_{jm}(k,t), \quad (15)$$

where the electric part is (Eq. 4.7 of Ref. 4)

$$\partial_t Q_{jm}(0,t) = \sqrt{4\pi j} \int d^3\mathbf{r}\ r^{j-1}\mathbf{Y}_{j,j-1,m}(\hat{\mathbf{r}})^\dagger \mathbf{J}(\mathbf{r},t), \quad (16)$$

and $T_{jm}(k,t)$ is the toroidal part.

It can be seen from Eqs. (14) to (16) that the integrand that defines $T_{jm}(k,t)$ must contain the $j_{j+1}(kr)\mathbf{Y}_{j,j+1,m}(\hat{\mathbf{r}})$ contribution plus the $j_{j-1}(kr)\mathbf{Y}_{j,j-1,m}(\hat{\mathbf{r}})$ contribution except for the first term in the small argument expansion of $j_{j-1}(kr)$, which is of order r^{j-1} and has been split up. This splitting corresponds to the one we have performed in Eq. (13). It causes the appearance of out of shell components in both electric and toroidal parts.

As mentioned in the main text and explained at the end of Sec. III in Ref. 1, the spherical Bessel functions inside the spatial integrals act as a filter that completely reject the out of shell ($|\mathbf{p}| \neq \omega/c$) components of the current. After the split, the term proportional to $(kr)^{j-1}$ by itself does not provide such rejection. This can be appreciated in the first line of Eq. (13): $r^{j-1}Y^*_{j-1\overline{m}}(\hat{\mathbf{r}})$ is a frequency independent function which cannot remove the $|\mathbf{p}| \neq \omega/c$ components present in $\mathbf{J}_\omega(\mathbf{r})$. Multiplication by a factor of k^{j-1} does not change this. Since a^ω_{jm} are physically measurable quantities without out of shell components, the presence of $|\mathbf{p}| \neq \omega/c$ components in the electric part implies their presence in the toroidal part with opposite sign, as it is obvious from Eq. (13).

The out of shell components do not couple to the electromagnetic field, and therefore preclude the independent physical measurement of the electric and toroidal parts.

IV. AN ALTERNATIVE DEFINITION OF THE TOROIDAL MULTIPOLES

In this appendix we first show that the definition of toroidal multipoles recently given in [6, Box 2] is different from the original definition in [3, 4]. Both definitions involve the split of the multipoles of electric parity into two parts, but the splits are different in [6, Box 2] and [3, 4]. We demonstrate this by showing that the well known expression of the toroidal dipole in the limit of small source [4, Eq. 2.11] cannot be recovered from the definition of [6, Box 2]. Some terms are missing. We then also show that the missing terms are contained in a coefficient which is explicitly excluded from the definition of toroidal multipoles in [6, Box 2]. The derivations in this appendix recover one of the results from the main text: The toroidal dipole is just the next to leading order term in the small source expansion of the exact electric dipole. The difference is that here the result is obtained directly in coordinate ($\mathbf{r}$) space, while Eq. (4) from the main text was obtained in [1] by first going to momentum ($\mathbf{p}$) space, and then going back to $\mathbf{r}$ space. Finally, we show that, in this alternative definition, both parts contain longitudinal terms, which render them non-separable.

We start by examining the definitions in [6, Box 2]

$$\begin{aligned}
\mathbf{E}_{\text{sca}}(\mathbf{r}) &= \frac{4\pi k^2}{c}\sum_{j,m}\left[Q_{jm}\mathbf{\Psi}_{jm}(\mathbf{r}) + M_{jm}\mathbf{\Phi}_{jm}(\mathbf{r}) + T_{jm}\mathbf{\Psi}_{jm}(\mathbf{r})\right], \\
Q_{jm} &= \frac{c}{\sqrt{j(j+1)}}\int d^3\mathbf{r}\ \rho_\omega(\mathbf{r})Y^*_{jm}(\hat{\mathbf{r}})\frac{d}{dr}\left[rj_j(kr)\right], \\
M_{jm} &= \frac{1}{i\sqrt{j(j+1)}}\int d^3\mathbf{r}\ \left[\nabla\cdot(\mathbf{r}\times\mathbf{J}_\omega(\mathbf{r}))\right]Y^*_{jm}(\hat{\mathbf{r}})j_j(kr), \\
T_{jm} &= \frac{k}{\sqrt{j(j+1)}}\int d^3\mathbf{r}\ \left[\mathbf{r}\cdot\mathbf{J}_\omega(\mathbf{r})\right]Y^*_{jm}(\hat{\mathbf{r}})j_j(kr),
\end{aligned} \quad (17)$$

where $\mathbf{E}_{\text{sca}}(\mathbf{r})$ is the field produced by the sources, and $\mathbf{\Psi}_{jm}(\mathbf{r})$ and $\mathbf{\Phi}_{jm}(\mathbf{r})$ are the multipolar fields of electric and magnetic parity, respectively. The Q_{jm} are said to be charge excitations yielding electric multipoles, the M_{jm} transverse (w.r.t $\mathbf{r}$) current excitations yielding magnetic multipoles, and the T_{jm} radial current excitations yielding toroidal multipoles.

We first note that Q_{jm} and T_{jm} are both multiplying the same multipolar field of electric parity $\mathbf{\Psi}_{jm}(\mathbf{r})$. This implies that, together, Q_{jm} and T_{jm} must completely determine the multipolar coefficients of electric parity. This can be readily checked by setting the magnetic currents to zero in Jackson's [7, Eq. 9.167] expression for the exact multipoles of electric parity $a_E(j,m)$, namely:

$$a_E(j,m) = \frac{k^2}{i\sqrt{j(j+1)}}\times \int d^3\mathbf{r}\ Y^*_{jm}(\hat{\mathbf{r}})\left\{c\rho_\omega(\mathbf{r})\frac{d}{dr}\left[rj_j(kr)\right] + ik\left[\mathbf{r}\cdot\mathbf{J}_\omega(\mathbf{r})\right]j_j(kr)\right\}. \quad (18)$$

It is clear from Eq. (17) that Q_{jm} corresponds to the first term of the sum in Eq. (18), and iT_{jm} to the second term. The sum $Q_{jm}+iT_{jm}$ determines $a_E(j,m)$. The definition of T_{jm} in Eq. (17) involves a split of the $a_E(j,m)$ into two parts. We now show that it is a different split from the one in the original definition of toroidal multipoles [3, 4].

Let us use iT_{1m}

$$iT_{1m} = \frac{ik}{\sqrt{2}} \int d^3\mathbf{r} \;\; [\mathbf{r} \cdot \mathbf{J}_\omega(\mathbf{r})] \, Y_{1m}^*(\hat{\mathbf{r}}) j_1(kr), \tag{19}$$

to attempt to recover the toroidal dipole in the small source approximation [4, Eq. 2.11],

$$\mathbf{t}_1^\omega = \int d^3\mathbf{r} \; \frac{1}{10} \left\{ [\mathbf{r} \cdot \mathbf{J}_\omega(\mathbf{r})] \, \mathbf{r} - 2r^2 \mathbf{J}_\omega(\mathbf{r}) \right\}, \tag{20}$$

and which follows from the original definition ([3, Eq. 39], [4, Eq. 2.11]).

We start by arranging the three components corresponding to $m = \{1, 0, -1\}$ into a vector,

$$i\mathbf{T}_1^{\text{sph}} = i \begin{bmatrix} T_{11} \\ T_{10} \\ T_{1-1} \end{bmatrix} = \frac{ik}{\sqrt{2}} \int d^3\mathbf{r} \;\; [\mathbf{r} \cdot \mathbf{J}_\omega(\mathbf{r})] \begin{bmatrix} Y_{11}^* \\ Y_{10}^* \\ Y_{1-1}^* \end{bmatrix} j_1(kr), \tag{21}$$

and consider the correspondence between $Y_{1m}^*(\hat{\mathbf{r}})$ and $\hat{\mathbf{r}}$ in the spherical basis

$$\hat{\mathbf{r}} = \frac{\mathbf{r}}{|\mathbf{r}|} = \begin{bmatrix} \hat{r}_1 \\ \hat{r}_0 \\ \hat{r}_{-1} \end{bmatrix} = 2\sqrt{\frac{\pi}{3}} \begin{bmatrix} -Y_{1-1} \\ Y_{10} \\ -Y_{11} \end{bmatrix} = 2\sqrt{\frac{\pi}{3}} \begin{bmatrix} Y_{11}^* \\ Y_{10}^* \\ Y_{1-1}^* \end{bmatrix}. \tag{22}$$

Equation (21) is a vector in the spherical vector basis except that, when both $\mathbf{r}$ and $\mathbf{J}_\omega(\mathbf{r})$ are expressed in the spherical vector basis, the term $[\mathbf{r} \cdot \mathbf{J}_\omega(\mathbf{r})]$ should be written $\left[\mathbf{r}^\dagger \mathbf{J}_\omega(\mathbf{r})\right]$, where $\mathbf{r}^\dagger$ denotes the hermitian conjugate of the position vector $\mathbf{r}$. This is due to the fact that, in the spherical vector basis, $\mathbf{r}$ has complex components. In the Cartesian basis used in [6, Box 2] and Eq. (20), $\mathbf{r}$ is real valued and the dot product $\left[\mathbf{r}^\dagger \mathbf{J}_\omega(\mathbf{r})\right]$ can be written as $[\mathbf{r} \cdot \mathbf{J}_\omega(\mathbf{r})]$. In this appendix we will use the Cartesian basis from now on. We can change $\mathbf{T}_1^{\text{sph}}$ to the Cartesian basis by multiplying $\mathbf{T}_1^{\text{sph}}$ itself, and the other vectors involved in the expression with the change of basis matrix

$$\begin{bmatrix} a_x \\ a_y \\ a_z \end{bmatrix} = \begin{bmatrix} \frac{-1}{\sqrt{2}} & 0 & \frac{1}{\sqrt{2}} \\ \frac{-i}{\sqrt{2}} & 0 & \frac{-i}{\sqrt{2}} \\ 0 & 1 & 0 \end{bmatrix} \begin{bmatrix} a_1 \\ a_0 \\ a_{-1} \end{bmatrix}. \tag{23}$$

After using Eq. (22) and changing the basis, the Cartesian expression reads:

$$i\mathbf{T}_1 = \frac{ik}{\sqrt{2}} \int d^3\mathbf{r} \;\; [\mathbf{r} \cdot \mathbf{J}_\omega(\mathbf{r})] \, \hat{\mathbf{r}} \sqrt{\frac{3}{\pi}} \frac{1}{2} j_1(kr). \tag{24}$$

We now use $j_1(kr) \approx kr/3$ in the limit of small kr, to approximate Eq. (24) by

$$\begin{aligned} i\mathbf{T}_1 &\approx \frac{ik}{\sqrt{2}} \int d^3\mathbf{r} \;\; [\mathbf{r} \cdot \mathbf{J}_\omega(\mathbf{r})] \, \hat{\mathbf{r}} \sqrt{\frac{3}{\pi}} \frac{1}{2} \frac{kr}{3} \\ &= \frac{ik^2}{\sqrt{2}} \sqrt{\frac{3}{\pi}} \int d^3\mathbf{r} \; \frac{[\mathbf{r} \cdot \mathbf{J}_\omega(\mathbf{r})] \, \mathbf{r}}{6}. \end{aligned} \tag{25}$$

Equation (25) cannot reproduce Eq. (20) because the $r^2 \mathbf{J}_\omega(\mathbf{r})$ terms present in Eq. (20) are missing in Eq. (25). The definition of T_{jm} in [6, Box 2] is hence different from the original definitions of the toroidal multipoles in [3, 4].

We now show that the missing terms are contained in Q_{1m}: They are the terms of second lowest order in the small source approximation of Q_{1m}. This reproduces our results from the main text.

We start with

$$Q_{1m} = \frac{c}{\sqrt{2}} \int d^3\mathbf{r} \;\; \rho_\omega(\mathbf{r}) Y_{1m}^*(\hat{\mathbf{r}}) \frac{d}{dr} [r j_1(kr)], \tag{26}$$

take the same steps as before regarding vectors and basis,

$$\mathbf{Q}_1 = \frac{c}{\sqrt{2}} \int d^3\mathbf{r} \;\; \rho_\omega(\mathbf{r}) \hat{\mathbf{r}} \sqrt{\frac{3}{\pi}} \frac{1}{2} \frac{d}{dr} [r j_1(kr)], \tag{27}$$

and use the continuity equation $\rho_\omega(\mathbf{r}) = \nabla \cdot \mathbf{J}_\omega(\mathbf{r})/(ikc)$, to obtain:

$$\mathbf{Q}_1 = \frac{-i}{k\sqrt{2}} \int d^3\mathbf{r} \;\; [\nabla \cdot \mathbf{J}_\omega(\mathbf{r})] \, \hat{\mathbf{r}} \sqrt{\frac{3}{\pi}} \frac{1}{2} \boxed{\frac{d}{dr} [r j_1(kr)]}. \tag{28}$$

We now consider the term in the shaded box, which, using the derivative of spherical Bessel functions[1] can be written as

$$\frac{d}{dr} [r j_1(kr)] = j_1(kr) + \left\{ \frac{kr}{3} [j_0(kr) - 2j_2(kr)] \right\}. \tag{29}$$

We now take terms up to order $(kr)^3$ in the small argument approximation of the spherical Bessel functions of Eq. (29):

$$\begin{aligned} j_0(kr) &\approx 1 - \frac{(kr)^2}{6}, \\ j_1(kr) &\approx \frac{kr}{3} \left(1 - \frac{(kr)^2}{10}\right), \\ j_2(kr) &\approx \frac{(kr)^2}{15}, \end{aligned} \tag{30}$$

to obtain the approximate expression

$$\begin{aligned} &\frac{d}{dr} [r j_1(kr)] \approx \\ &\frac{kr}{3} \left(1 - \frac{(kr)^2}{10}\right) + \left\{ \frac{kr}{3} \left[1 - \frac{(kr)^2}{6} - 2\frac{(kr)^2}{15}\right] \right\}. \end{aligned} \tag{31}$$

We now plug Eq. (31) into the right hand side of Eq. (28)

$$\begin{aligned} \mathbf{Q}_1 \approx &\frac{-i}{k\sqrt{2}} \sqrt{\frac{3}{\pi}} \frac{1}{2} \int d^3\mathbf{r} \;\; [\nabla \cdot \mathbf{J}_\omega(\mathbf{r})] \, \hat{\mathbf{r}} \frac{2(kr)}{3} + \\ &\frac{-i}{k\sqrt{2}} \sqrt{\frac{3}{\pi}} \frac{1}{2} \int d^3\mathbf{r} \;\; [\nabla \cdot \mathbf{J}_\omega(\mathbf{r})] \, \hat{\mathbf{r}} \frac{(kr)^3}{3} \left(-\frac{1}{10} - \frac{1}{6} - \frac{2}{15}\right), \end{aligned} \tag{32}$$

[1] $\frac{d}{dx} j_l(x) = \frac{1}{2l+1} \left(l j_{l-1}(x) - (l+1) j_{l+1}(x)\right)$

and reduce it to

$$\mathbf{Q}_1 \approx \frac{-i}{\sqrt{6\pi}} \int d^3\mathbf{r}\ \left[\nabla \cdot \mathbf{J}_\omega(\mathbf{r})\right] \mathbf{r} + \\ i\frac{k^2}{\sqrt{2}}\sqrt{\frac{3}{\pi}} \int d^3\mathbf{r}\ \frac{\left[\nabla \cdot \mathbf{J}_\omega(\mathbf{r})\right] r^2\mathbf{r}}{15}, \tag{33}$$

Using steps similar to those in [7, Eq. 9.14], the first line of Eq. (33) can be readily brought to the familiar form of the approximate electric dipole for small sources:

$$\frac{i}{\sqrt{6\pi}} \int d^3\mathbf{r}\ \mathbf{J}_\omega(\mathbf{r}). \tag{34}$$

This lowest order term is precisely the one separated by Dubovik [3, 4] in his split of the exact multipoles of electric parity between what is referred to as the "electric" part, which is this term in [7, Eq. 9.170], and the toroidal part, which are the higher order terms.

Incidentally, reversing the use of the continuity equation in the first line of Eq. (33) recovers the first line in [7, Eq. 9.170].

We are now interested in the next to leading order terms of $\mathbf{Q}_1$, which are shaded in the second line of Eq. (33). We now show that, when summed to Eq. (25), the toroidal dipole is recovered. The key step is to use the divergence theorem in the integration by parts of Eq. (33). To that end we first consider the following steps, where n and k run over $\{1,2,3\}$ and δ_{nk} is the Kronecker delta:

$$\nabla \cdot \left(\mathbf{J}r^2 r_k\right) = \sum_n \left[\left(\partial_n J_n\right) r^2 r_k + J_n \partial_n (r^2 r_k)\right] = \\ \sum_n \left[\left(\partial_n J_n\right) r^2 r_k + J_n (2 r_n r_k + r^2 \delta_{nk})\right] = \\ \left(\nabla \cdot \mathbf{J}\right) r^2 r_k + 2\left(\mathbf{r}\cdot\mathbf{J}\right) r_k + J_k r^2. \tag{35}$$

Since $\mathbf{J}_\omega(\mathbf{r})$ is, by assumption, bounded in space, the divergence theorem can be used to show that

$$\int d^3\mathbf{r}\ \nabla \cdot \left[\mathbf{J}_\omega(\mathbf{r}) r^2 r_k\right] = 0 \text{ for all } k. \tag{36}$$

The divergence theorem [7, p. 36]

$$\int_V d^3\mathbf{r}\ \nabla \cdot \mathbf{A} = \oint_S \mathbf{A} \cdot \hat{\mathbf{r}}\ dS \tag{37}$$

relates the integral of the divergence of any well-behaved vector field $\mathbf{A}$ over a volume V to the flux through the surface boundary of V. For our purposes we set $\mathbf{A} = \mathbf{J}_\omega(\mathbf{r}) r^2 r_k$, and choose V as a spherical volume enclosing the sources, so that $\mathbf{J}_\omega(\mathbf{r}) r^2 r_k$ is zero on its surface boundary. Then, the right hand side of Eq. (37) vanishes.

Therefore, using Eq. (35) for $k = \{1,2,3\}$ we obtain

$$\int d^3\mathbf{r}\ \left[\nabla \cdot \mathbf{J}_\omega(\mathbf{r})\right] r^2\mathbf{r} = \\ -2\int d^3\mathbf{r}\ \left[\mathbf{r}\cdot\mathbf{J}_\omega(\mathbf{r})\right]\mathbf{r} - \int d^3\mathbf{r}\ r^2 \mathbf{J}_\omega(\mathbf{r}), \tag{38}$$

whose left hand side appears in Eq. (33). After substituting Eq. (38) into Eq. (33) we get:

$$i\frac{k^2}{\sqrt{2}}\sqrt{\frac{3}{\pi}} \int d^3\mathbf{r}\ \frac{1}{15}\left\{-2\left[\mathbf{r}\cdot\mathbf{J}_\omega(\mathbf{r})\right]\mathbf{r} - r^2\mathbf{J}_\omega(\mathbf{r})\right\}. \tag{39}$$

Let us recall that Eq. (39) are the next to leading order terms in the small source expansion of Q_{1m}. After summing them to Eq. (25)

$$i\frac{k^2}{\sqrt{2}}\sqrt{\frac{3}{\pi}} \int d^3\mathbf{r}\ \left(-\frac{2}{15}+\frac{1}{6}\right)\left[\mathbf{r}\cdot\mathbf{J}_\omega(\mathbf{r})\right]\mathbf{r} - \frac{1}{15} r^2\mathbf{J}_\omega(\mathbf{r}) = \\ i\frac{k^2}{\sqrt{6\pi}} \int d^3\mathbf{r}\ \frac{1}{10}\left\{\left[\mathbf{r}\cdot\mathbf{J}_\omega(\mathbf{r})\right]\mathbf{r} - 2r^2\mathbf{J}_\omega(\mathbf{r})\right\}, \tag{40}$$

we recover the exact form of the toroidal dipole in Eq. (20).

This derivation also recovers a result from the main text: The toroidal multipoles are just higher order terms in the small source approximation of the exact multipoles of electric parity.

To conclude, we note that, according to Eq. (28), which contains $\nabla \cdot \mathbf{J}_\omega(\mathbf{r})$, $\mathbf{Q}_1$ depends on the longitudinal degrees of freedom of $\mathbf{J}_\omega(\mathbf{r})$. This follows from the $\mathbf{r}$ domain to $\mathbf{p}$ domain correspondence

$$\nabla \cdot \mathbf{J}_\omega(\mathbf{r}) \to i\mathbf{p}\cdot\mathbf{J}_\omega(\mathbf{p}), \tag{41}$$

which makes clear that the c_{jm}^{ω} coefficients multiplying the longitudinal multipoles contribute to $\mathbf{Q}_1$ [see Eqs. (2) and (3)].

Since the sum $Q_{jm}^{\omega} + iT_{jm}^{\omega}$ is equivalent to the fully transverse a_{jm}^{ω}, it then follows that the longitudinal dependence introduced by $\mathbf{Q}_1$ must be canceled by $i\mathbf{T}_1$. The split introduces longitudinal components in both parts, which renders them non-separable according to the discussion in the main text (see the section entitled "Splits without out of shell components").

[1] Ivan Fernandez-Corbaton, Stefan Nanz, Rasoul Alaee, and Carsten Rockstuhl, "Exact dipolar moments of a localized electric current distribution," Opt. Express **23**, 33044–33064 (2015).

[2] A. J. Devaney and E. Wolf, "Multipole expansions and plane wave representations of the electromagnetic field," J. Math. Phys. **15**, 234–244 (1974).

[3] V. M. Dubovik and A. A. Cheshkov, "Multipole expansion

in classical and quantum field theory and radiation," Sov. J. Part. Nucl. **5**, 318–337 (1974).

[4] V. M. Dubovik and V. V. Tugushev, "Toroid moments in electrodynamics and solid-state physics," Phys. Rep. **187**, 145–202 (1990).

[5] E. Radescu and G. Vaman, "Exact calculation of the angular momentum loss, recoil force, and radiation intensity for an arbitrary source in terms of electric, magnetic, and toroid multipoles," Phys. Rev. E **65**, 046609 (2002).

[6] N. Papasimakis, V. A. Fedotov, V. Savinov, T. A. Raybould, and N. I. Zheludev, "Electromagnetic toroidal excitations in matter and free space," Nat. Mater. **15**, 263–271 (2016).

[7] John David Jackson, *Classical Electrodynamics* (Wiley, 1998).
